# Prophylactic Odontotomy: The Cutting into the Tooth for the Prevention of Disease*Read before the Academy of Stomatology, Philadelphia, October 24, 1922; reprinted with *Discussion* from the *Dental Cosmos* for March, 1923.

**Published:** 1923-05

**Authors:** Thaddeus P. Hyatt

**Affiliations:** New York, N. Y., Dental Director, Metropolitan Life Insurance Company


### PROPHYLACTIC ODONTOTOMY: THE CUTTING INTO THE TOOTH FOR THE PREVENTION OF DISEASE *

BY THADDEUS P. HYATT, D. D. S., NEW YORK, N. Y.

Dental Director, Metropolitan Life Insurance Company

### INTRODUCTION

*Mr. President and Friends:*

I feel an apology is due you. It is usual to expect an essayist to present something new at a meeting of this nature. Some new discovery to account for the prevalence of decay. Some new invention whereby all root canals can be permanently and perfectly filled and all possible systemic disturbances avoided. Some new formula that will cure pyorrhea and absolutely prevent its occurrence and reoccurrence. In other words we look forward to learning something new, something we did not know before. Tonight I am simply bringing before you facts and figures of things as they are today and asking for your attention and consideration.

Conditions ofttimes become so unusual that we accept them as being quite natural and unchangeable.[1] We cease

---

* Read before the Academy of Stomatology, Philadelphia, October 24, 1922; reprinted with *Discussion* from the *Dental Cosmos* for March, 1923.

[1] *An example:* One thousand and sixty-one children of pre-school age were examined for physical defects, by a number of welfare organizations in New York City. These are the defects found:

to wonder at or to question the reasonableness of their presence and we fail to devote much time or thought to the possibility of their removal. Rather do we devote our abilities and inventive ingenuity to the acceptance of these conditions and to the treatments required by their presence. In other words, our attitude is analogous to a body of people living in a swamp, who devote their entire time to inventing ways and means of living as comfortably as possible in the swamp. They do not study nor do they consider the possibility of draining the swamp, and thus remove all necessity for gutta-percha encapsulation of their feet, seamless gold for waterproof caps or movable-removable gowns that will not impede their graceful motions while wading through the mud or slush.

My purpose in drawing your attention to present conditions is not with the thought of offering any panacea for the prevention of all dental troubles but rather in the hope of being able to suggest some procedure *which may very largely, if not entirely,* remove a state now so prevalent and *tide us over* to that period when right liv-

---

| | Per Cent. |
|---|---|
| Hypertrophied tonsils | 26.3 |
| Defective nasal breathing | 23.1 |
| Malnutrition (3 and 4) | 19.2 |
| Defective teeth | 72.6 |
| Pulmonary disease | 1.12 |
| Organic cardiac | .94 |
| Nervous disease | .66 |
| Orthopedic disease | 1.12 |

The physician making this report says: "The outstanding features are that the predominating defects found were: Hypertrophied tonsils, defective nasal breathing and malnutrition (3 and 4)." These three added together make only 68.6 per cent. while dental defects are 72.6 per cent. I wrote to the society publishing this report asking if they could explain why the doctor made no mention of dental defects.

This is the reply: "I do not think Dr. —— means in his own part of the report to slight the importance of defective teeth. I judge he feels defective teeth are so common and found in so many children, that they are not given special mention." We know tonsils, nasal breathing and nutrition are affected for weal or woe by dental conditions and because dental defects are so common they are not given special attention.

ing and correct eating will make dentists and physicians an unnecessary blessing.

With this introduction, Mr. President, permit me to present my paper.

---

Today the dental profession is confronted with one of the most important problems affecting the health, efficiency and happiness of their fellow men.

During the past twenty years we have learned that health is seriously impaired by inefficient mastication and even life is endangered by septic mouth conditions.

The laity and the medical profession properly look to us for the answer to the question: What can be done?

When we view mouth conditions in the industries and in our public schools, as have been shown by examinations made during the late draft, the situation is truly appalling.

The fact that there is an average of five cavities in the mouth of every man, woman and child in the United States does not illustrate the real significance of the situation.

Many millions of these persons have teeth with fillings that have not been included in the estimate of cavities given. Many millions have lost teeth, and their loss has brought about traumatic occlusion, with gingivitis and other malconditions of the soft tissues.

Many millions of fillings are covering diseased and nonvital pulps, while other countless millions of fillings are covering decayed dentin, which is being slowly absorbed in the system through the veins and lymphatics of the tooth pulp.

Large sums of money are being spent and much valuable time is being devoted to scientific research work to determine whether teeth with nonvital pulps are as dangerous to health as many believe and figures seem to prove.

Much thought and study is being given to the consideration of the best technique for root-canal work, vulcanite dentures, stationary, movable and removable bridge work, cast gold inlays, etc.

We might paraphrase and say: Inlays may come and fillings may go, but decay goes on forever.

In our efforts to explain our inability to cope with the conditions that confront us, we offer many theories which seem reasonable; but they do not stop, nor do they prevent, dental caries.

One of the theories attracting the attention of our profession today is the question of diet and malnutrition. There can be no doubt that white flour, white sugar and soft food are the curses of present-day civilization. But even if it were possible to change our mode of eating and the foods eaten, it would probably take several generations before any marked change in the structure of the teeth would be noticed.

If the dental profession is to approach this subject in a practical way, it must be upon a platform of that which is actually possible for us to accomplish and then present this view in such a manner that it will be accepted and put into effect.

Up to the present time our attitude toward dental decay is to wait until decay has appeared. The dental profession and the public have not considered the advisability of operating upon sound teeth. We have not studied what advantages there may be if we should practice what would be analogous in its effects to vaccination or inoculation. According to the procedure which I shall present this evening this dental vaccination or inoculation would mean the cutting and placing of fillings in certain surfaces of perfectly sound teeth *before* decay has started.

We fully understand that the people should be educated along the lines of proper diet, particularly for expectant mothers and young children. Such a campaign, however, will take many years before any diminution of susceptibility to dental caries is brought about. What are we to do during this time? Allow these millions of teeth that have already erupted to become decayed before attempting to give them any attention? Must we follow along the lines of reparative measures *after* decay has started? We know that up to now this has largely been a failure.

With due respect to the wonderful advancement made in dental science and with a keen appreciation of the really marvelous skill and technique developed in recent years, we all know that destruction of the dental organs is as prevalent now as it was twenty or thirty years ago.

In presenting or advocating a new dental procedure, particularly one which is a decided change from the present accepted idea, it is advisable to give due consideration to the situation that faces the dental profession.

Today it is a condition that confronts us and not a theory. What is this condition?

In an examination of 5,000 adults employed in two different industries, which include foreign as well as native-born men and women, we find 47 per cent. of the first permanent molars, 17 per cent. of the second permanent molars and 14 per cent. of the second premolars lost.

Let us examine this condition carefully from a health point of view and ascertain what this condition really implies. This necessitates the consideration of masticating efficiency.

When an upper or lower molar is lost, the masticating efficiency of the opposing molar is destroyed. Masticating efficiency is dependent upon the first and second molars and the second premolars. Among these 5,000 employes we find there is a loss of 15,757 masticating teeth, causing the impairment of 15,757 other masticating teeth making a total loss of 31,514 masticating teeth in the mouths of 5,000 persons. We thus find that over 50 per cent. of masticating efficiency has been lost or destroyed.[2]

---

[2] These figures should not be taken literally, but generally, as they are not scientifically nor mathematically correct, for it might be possible that the first superior right molar and the first lower right molar were missing. If this were so it would be incorrect to double them and call it the loss of four masticating teeth.

This does not take into consideration the impairment of the masticating efficiency of those teeth which have moved out of their place through the loss of adjacent teeth and thus do not come into proper masticating occlusion with their opposing tooth. Nor does it take into consideration the loss of masticating efficiency brought about by poorly-constructed occlusal fillings placed in teeth that were badly decayed.

We must realize that while we have spoken of the loss of the masticating efficiency of a certain number of teeth, we have not presented figures to show what percentage of the 5,000 persons have lost their masticating efficiency. In all probability 70 per cent. of the persons have not the complete masticating efficiency required for the maintenance of full physical health and happiness. When we take into consideration the inefficiency of the balance of the teeth, such as the anterior teeth, for masticating purposes, we must realize the significance and importance of these figures when viewed from the standpoint of health.[3] We must also consider the septic conditions brought about by such dental decay.

In an examination made in 1916 of 1,462 employes of the Metropolitan Life Insurance Company between the ages of 17 and 54, over 22 per cent. of the first molars were lost. This naturally brought about the loss of 44 per cent. of the masticating efficiency of teeth alone.[2]

In an examination of 8,069 high school pupils, we find upward of 15 per cent. of first molars lost and 10 per cent. badly decayed; 2 per cent. of second molars lost and 2 per cent. badly decayed; 2 per cent. of second premolars lost and 2 per cent. badly decayed. This will give us the loss of 22 per cent. of masticating efficiency in children before they reach the age of manhood.[2]

It is not my intention, nor is it necessary for me to go into the theory of dental caries or to give a résumé of the

---

[3] HARTZELL, THOMAS B.: "Ten Years to Life," *Journ. American Dental Association*, October, 1922, p. 837.

bacteriology of decay. We are all familiar with the investigations that have been made along these lines. Suffice it to call your attention to the fact that such investigators as Miller, Williams, Pickerill, Bunting, Goadby, Black and others are all agreed that no matter what are the facts or factors that produce dental caries, the pits and fissures in molars and premolars are the places most susceptible to decay.

"Imperfections of the teeth, such as pits, fissures, rough or uneven surfaces and bad forms of interproximate contact, are causes of caries only in the sense of giving opportunity for the action of the causes that induce caries." [4]

It is interesting to find that even rocks suffer from similar imperfections. In the *Literary Digest* for September 23, 1922, page 23, we read: "Granite is regarded as one of the firmest foundations. It is liable, however, to blind joints, invisible planes on which there has been no actual parting, but the minerals have been strained and are ready to react to forces of decay. The engineer can not discover blind joints. Investigation by the microscope alone can demonstrate whether or not they are present. They seek to excavate to foundation rocks which show no open joints, or to seal visible joints with cement."

However, in most of the writings on the subject of dental caries and the loss of teeth through caries, consideration does not seem to have been given to the numerical differences in susceptibility to decay of the different surfaces of the teeth. There has been but little research work done along these lines. The only articles that I know of which show this difference is one by Doctor Butler of Washington, D. C., entitled: "The Fate of the First Molar," and one by myself published in the *Dental Cosmos* for April, 1920, and called "Report of an Examination Made of Two Thousand One Hundred and One High School Pupils."

---

[4] BLACK, G. V.: "Physical Characters of the Human Teeth," *Dental Cosmos*, 1895, Vol. xxxvii, p. 416.

If we show that one surface has a susceptibility far exceeding all other surfaces, a procedure may be suggested which may prove to be the real preventive dentistry we desire. It may also explain the reason why our present methods and procedures are not productive and can not be productive of better results.

While granting that malnutrition has much to do with increasing the susceptibility of teeth to dental caries, this does not alter the fact that it is doubtful if malnutrition alone will bring about decay in already formed and erupted teeth without other factors being present. In other words, malnutrition *alone* will probably not produce decay in teeth already present in the mouth.

Proper nutrition is necessary for health. Right living, fresh air, proper exercise, are all understood as being necessary and desirable, so they are accepted and taken for granted without any dispute or discussion. While the loss of any of these desirable conditions undoubtedly makes our work the harder, they do not entirely remove all possibilities on our part to practice preventive dentistry and in a very great measure to save and retain most of the masticating dental organs.

In considering the procedure which I desire to present for your consideration there are several important facts which I believe are practically accepted by all members of our profession and which must be borne in mind as I read my paper. These are as follows:

First. In its relation to decay, a tooth is divided into two parts: (*a*) That part which is susceptible to decay, and (*b*) that part which is practically immune to decay.

Second. Decay starts at the periphery of the tooth and travels pulpward.

Third. The influence of decay precedes decay and is invisible to the naked eye. Its presence is known from microscopic study of decayed teeth. Its entire removal is never certain and at the present time there is not any positive or scientific means of knowing if we have removed it all.

Fourth. The presence of the influence of decay under the most perfect filling is always dangerous and it is an ever-potent possibility for the beginning of new destruction to the teeth.

That certain parts of a tooth are practically immune to decay has been clearly shown by Doctor Black and this has led to the practice of "Extension for Prevention" or, in other words, extending the walls of the cavity out from the area of susceptibility into the area of immunity.

The areas of susceptibility can be divided into: susceptibility because of location and susceptibility because of anatomical construction.

Examples of susceptibility because of location are the interproximal surfaces.

Examples of susceptibility because of anatomical construction are the fissures and pits which are mostly found on the molars and premolars.

While malnutrition may increase the susceptibility of teeth to decay, it is doubtful if it makes susceptible those parts of the teeth which are known as being immune to decay. In other words, the immunity of certain parts of the tooth is not endangered by malnutrition. The only danger to these immune parts is the extension of decay from the already susceptible areas.

Reduction of vital resistance through malnutrition increases the rapidity of tooth destruction in the areas of susceptibility and from here it may extend and destroy the entire tooth.

Anatomically the occlusal pits and fissures of molars and premolars are susceptible to decay, because of faulty formation or the inability of keeping them absolutely free from the lodgment of bacteria and food débris.

Allow me, at this place, to call your attention to these very interesting quotations from Doctor Grieves' paper, "A Preliminary Study of Gross Maxillary and Dental Defects in Three Hundred Rats on Defective and Deficient

Diet."[5] Under the heading "Caries-like Defects in Molar Group" in Table III is shown the incidence of these defects in rats restricted to several types of defective diet discussed in the paper.

These lesions which always proceed from without inward do not result from hypoplastic enamel. They are not absorptions by dental pulp function but have every microscopic characteristic of caries. That the defects which occurred in the teeth of these experimental rats were comparable to those found in human dental caries is seen in the minute and slowly enlarging initial lesion in any deep enamel sulcus; the rapid dental invasion undermining enamel walls; * * * The majority of lesions arise in distal sulci of the first and central sulci of the second molars. These sulci are much deeper than those in the third molars and caries-like defects may result from food retention. * * * The incisors even when hypoplastic or dystrophic are not involved by caries-like defects. Possibly this may be due to (1), the simple tooth form and constant use in gnawing, which prevents food débris retention; (2), to the excellent resistance maintained by persistent enamel organs and pulps.

Many contend that if an expectant mother receive proper dental attention and proper diet this occlusal susceptibility can be and will be greatly reduced. This may be true. At present it is a theory. While we are testing out and proving the correctness of this theory, what is to happen to the many millions of molars and premolars already erupted?

As I have said before, it is a condition that confronts us. How bad is this condition regarding the number of occlusal cavities as compared with cavities on other surfaces, I now propose to show.

In an examination of 2,101 girls in one of the public schools, a study was made of the location of cavities in

[5] *Journal of the National Dental Association*, June, 1922, Vol. ix, p. 486.

first molars. Roughly speaking it is found that occlusal cavities are three times as many as buccal cavities, five times as many as mesial cavities, seven times as many as distal cavities, nineteen times as many as lingual cavities and more than double the number of all cavities in all other surfaces added together.

Dr. H. B. Butler of the United States Health Service, made a study of 1,000 cavities in the first permanent molars. He found 877 were occlusal, 110 mesial, 8 distal, 3 buccal and 2 lingual. According to these figures, occlusal cavities are seven times the number of all cavities in all surfaces added together.

These figures should clearly show us that the point of attack for the prevention of dental caries is the occlusal surface.

TABLE I

*First Molars*

Percentage of those having cavities. Surface on which cavities are located. Percentage of teeth lost.

| | No. of Persons | First Molar | | | | | |
|---|---|---|---|---|---|---|---|
| | | Occlusal | Buccal | Lingual | Distal | Mesial | Lost |
| Rochester | 500 | 68 | 6 | 1 | 2 | 8 | 6 |
| New York City— | | | | | | | |
| Red Cross No. 1 | 633 | 44 | 1 | 2 | 6 | 4 | 13 |
| Red Cross No. 2 | 159 | 93 | 7 | 3 | 32 | 26 | 5 |
| Red Cross No. 3 | 931 | 86 | 1 | 2 | 31 | 30 | 9 |
| North Carolina | 21,577 | 85 | 8 | 2 | 3 | 4 | 8 |
| Dr. H. B. Butler | ... | 88 | 3 | 2 | 8 | 11 | ? |
| Oral Hygiene Committee of Greater New York | 8,068 | 62 | 5 | 2 | 16 | 18 | 15 |
| Girls' High School | 2,101 | 59 | 21 | 3 | 8 | 9 | 14 |
| Wayne School | 770 | 84 | 6 | 3 | 3 | 4 | 9 |
| Industrial | 5,000 | 75 | 14 | 14 | 17 | 23 | 47 |
| Total | 39,739 | 744 | 72 | 34 | 126 | 137 | 126 |
| Average Percentage | ... | 74 | 7 | 3 | 13 | 14 | 14 |

TABLE II

*Second Molars and Premolars*

Percentage of those having cavities. Surface on which cavities are located. Percentage of teeth lost. Increase according to age.

| Ages | No. of Persons | Second Molars | | | | | | Second Premolars | | | | | |
|---|---|---|---|---|---|---|---|---|---|---|---|---|---|
| | | Occlusal | Buccal | Lingual | Distal | Mesial | Lost | Occlusal | Buccal | Lingual | Distal | Mesial | Lost |
| Between 7 and 14.. | 500 | 8 | 2 | 0 | 0 | 1 | 8 | 12 | 2 | 0 | 2 | 1 | 0 |
| " 14 and 19.. | 8,068 | 45 | 3 | 1 | 3 | 3 | 2 | 11 | 0.5 | 0.3 | 4 | 4 | 2 |
| " 17 and 55.. | 5,000 | 74 | 14 | 7 | 9 | 8 | 17 | 75 | 2 | 10 | 13 | 17 | 14 |

As dental decay always starts at the periphery, or on the outside of the tooth, and then travels inward toward the pulp *it must be blocked at the starting point* if it is to be combated successfully.

We must cut away this susceptible part of the tooth before decay has started and fill it with a material not susceptible to decay.

We must start our campaign for preventive dentistry on the outside of the tooth and not only on the inside of the body.

This campaign has practicability and possibilities for immediate action with the greatest promise of desirable results.

Cavity preparation and the procedure for fillings are thoroughly understood. It is not necessary for me to describe them. My purpose is to show you *why* we should cut away these occlusal fissures and fill them *before* decay has started, and not how to fill them. I do, however, suggest the following: As soon as these teeth have erupted so access can be had to the occlusal surface, prophylactic fillings should be placed there. This can be done by carefully cleaning and drying the fissures. A fine pointed explorer is used to aid in the cleansing. Wash off the surface with warm water and hydrogen dioxid. Then carefully dry the tooth. Cement, such as oxyphosphate of copper or silver cement, is then worked down into the fissure with a fine exployer and the surplus wiped off.

When the tooth has erupted sufficiently, apply a rubber dam. Make a class I cavity. The required outline form will bring the walls of the cavity within the area of immunity. Sufficient depth should be secured to obtain the required resistance. A slight undercut will give sufficient retention. Our work is simplified because there is no carious dentin to remove. If amalgam is used, it is advisable to burnish the filling at a subsequent sitting. This will secure a smooth finish and good marginal adaptation at enamel walls.

Why should we wait until decay has started? We know the difficulty of following decay to its ultimate extent or removing that part of the dentin which has been influenced by the presence of decay.

We know the difficulty of detecting the first stages of decay. Dr. J. Leon Williams has shown that "acid effects penetrate the entire thickness of the enamel before there is any sufficient breakdown of the surface to be detected with the finest exploring point.[6]

We also know that many times when we begin to excavate what we think is a small cavity we find it has extended far into the dentin in every direction. We know what will happen if every particle of decay is not removed. We do not know, however, how far the influence of decay has penetrated and affected the seemingly sound dentin.

On the other hand, if we cut into the fissures of a sound tooth, we shall not deal with decay in any form. With the rubber dam, clean instruments, thorough removal of all débris, the cavity will be clean and practically aseptic. A thin solution of carbolized resin or cavity varnish can be used to paint the surface of the cavity, after which it is thoroughly dried with warm air.

---

[6] Williams, J. Leon: *Journal of Dental Research*, 1919, Vol. i, p. 24.

Judgment is to be exercised in deciding whether the occlusal fissures of molars are to be followed to and on the buccal surface. As only 7 per cent. of buccal surfaces have cavities, it can readily be seen that the necessity for this extension is not very often needed.

Because susceptibility of the interproximal surface does not entirely depend upon the anatomical or faulty structure of the tooth as it is found upon the occlusal surface, we may, with clear conscience, very largely leave the care of this surface to the patient. We must, however, instruct and teach the patient how and why they should keep these interproximal surfaces particularly clean as well as give proper care to the mouth as a whole.

I feel that the occlusal surfaces of first and second molars and second premolars are our responsibility. Knowing as we do that approximately 70 to 75 per cent. will decay sooner or later; knowing too, that in many cases no amount of right cleansing will prevent their decaying, why should we not bravely and conscientiously meet the situation that confronts us and go to work and cut out and fill these pits and fissures in the occlusal surfaces of these teeth before decay has started, so they may have a fair chance to live in health and soundness and thus render to their owners the aid and service they were created to perform.

Some of us can do this in our private practice, but all should be sustained and supported in this work by the official indorsement of our profession so that this procedure may be adopted in all public dental work, such as in the public schools and elsewhere. This is necessary for the reason that for ages past the world at large and the dental profession have only thought it necessary to fill teeth after decay has started.

To advocate the filling of any part of a tooth before decay has commenced is such a change from the old idea of dental service, that I feel something more is necessary than the acceptance of this idea by individual dentists.

In concluding, I wish to express my thanks to the following persons and organizations who have assisted me in securing the figures that have enabled me to present these facts to your Society in such a way as to show the desirability, practicability and reasonableness for the procedure advocated:

Mr. George R. Bedinger, New York City; Dr. Harvey J. Burkhart, Rochester, N. Y.; Dr. Harry B. Butler, Washington, D. C.; Miss M. E. Colony, Manual Training High School, New York; Dr. G. M. Cooper, Director, State Board of Health, N. Carolina; Dr. Harold DeW. Cross, Boston, Mass.; Dr. Louis I. Dublin, Statistician, Metropolitan Life Ins. Co.; Mr. J. C. Gebhart, New York; Mr. Edward T. Hartman, Philadelphia, Pa.; Dr. Thomas P. Ryan, Minneapolis, Minn.; Miss Caroline Wollaston, Girls' High School, New York.

Organizations: Colgate Company, New York; Girls' High School, Physical Training Department, Brooklyn, N. Y.; New York Chapter American Red Cross; North Carolina Department of Health; Oral Hygiene Committee of Greater New York; Rochester Dental Dispensary, Rochester, N. Y.; Wayne School, Philadelphia, Pa.

BIBLIOGRAPHY

ANTHONY: "Consideration of the Dietetic Factor in the Problem of Dental Caries." *Dental Cosmos.* 1922.

BLACK: "An Investigation of the Physical Characters of the Human Teeth." *Dental Cosmos.* 1895.

BUNTING AND RICKERT: "Dental Caries." *Journal National Dental Association.* 1915.

BURNETT: "Human Pearls." 1908.

BUTLER: "The Fate of the First Molar." Public Health Reports. 1921.

ECKERMAN: "Dental Caries in Relation to Oral Osmosis." 1919.

FONES: "Mouth Hygiene." 1921.

GIVEN: "Dental Efficiency in the Navy," etc. *British Dental Journal.* 1922.

GOADBY: "Mycology of the Mouth." 1903.

GRIEVES: "Preliminary Study of Gross Maxillary and Dental Defects." etc. *Journal National Dental Association.* 1922.

HECHT: "Gateway of Health." 1921.

HOPKINS: "Care of the Teeth." 1902.

HOWE: "Report on Studies of the Effect of Vitamin-Deficient Diet Upon the Teeth." *Dental Cosmos.* 1921.

HYATT: "Report of an Examination of 2,101 High School Pupils." *Dental Cosmos.* 1920.

MELLANBY, WM.: "Influence of a Special Dietetic Factor on the Development of the Teeth and Jaws." *Dental Record,* London. 1920.

MUMMERY: "Summary of Existing Knowledge in Regard to Variation in Composition and Structure of Enamel," etc. *British Dental Journal.* 1922.

PICKERILL: "The Prevention of Dental Caries and Oral Sepsis." 1912.

ROSE: "Care of the Teeth and Mouth." 1900.

TANNER: "Mouth and Teeth." 1914.

WALLACE: "The Care and Prevention of Decay in Teeth." 1902.

WILLIAMS: "Contribution to the Study of Pathology of Enamel." *Dental Cosmos.* 1897.

### DISCUSSION

DR. E. T. DARBY. *Mr. President, Ladies and Gentlemen of the Academy:* There is probably no subject that is engaging the attention of the American people, and I might say the civilized people of the world today, more than the teeth. They are awakening to the fact that the teeth are of the utmost importance to their comfort and health and to the importance of their preservation. I have been very much interested in Doctor Hyatt's paper, because he has given us statistics that are authoritative. He has had ample opportunity in the Metropolitan Life Insurance Company of examining the teeth of 7,000 people employed in that building. His examinations there have apparently been most thorough, and added to his work there he has had the opportunity of examining hundreds of other mouths throughout the country, of which he has given us statistics. It is undoubtedly true that teeth are decaying more frequently than they did 3,000 or 4,000 years ago. If you will pardon me, I will give you my experience among the mummies in Egypt fifty years ago. It was my privilege to have been in Egypt during the Franco-Prussian War, when the Frenchman who had charge of the mummy pits of Sakkarah was locked up in Paris. An old sheik, who lived in a little hut in the desert, not far from the pits, was left in charge of them during the Frenchman's absence. I asked the dragoman if he thought I would be permitted to take possession of those pits, pull out the mummies, cut the bandages from their faces and examine the teeth. He thought not, unless I made it a great object to the man to go away and leave me with the dead. I suggested that if we put a sovereign over each eye he would not see what I was doing. The suggestion proved a good one and after standing about for a few moments he went to his hut and did not return. We brought out mummy after mummy, scores of them, cut the bandages from the heads and examined the teeth. They had been embalmed after one of the most expensive methods then known to the Egyptians and the bandaging had been most beautifully done. They had been buried for 3,000, perhaps 4,000, years and were in perfect condition as mummies. To my great surprise I did not find a single evidence of caries. In all those examined there was but a single tooth missing, that being an inferior bicuspid. There was great absorption of the alveolar process, which seems to indicate an abscess and perhaps necrosis.

I question if an examination of an equal number of civilized people today would reveal such a condition of the teeth.

It is undoubtedly true that teeth are more prone to caries than they were 3,000 years ago, and yet I am led to believe that the American people have better teeth than they did fifty or one hundred years ago. How much of this is due to the greater care given them and the service of the dentist I can not say.

I was specially interested in the statistics Doctor Hyatt gave us of the relative frequency of caries in the first and second molars and his method of preventing or arresting caries before it had done any damage to the tooth.

Many years ago Doctor Magitot of Paris examined 10,000 teeth (I take it they were extracted teeth) and he reported the following: Of first molars there were 3,350 decayed, of these 1,810 were inferior and 1,540 superior. Of second molars there were 1,736, of which 1,046 were in the inferior jaw and 690 in the superior. This table showed that the molars were more susceptible to decay than any other teeth in the mouth. I am quite sure every dentist would certify that his observation has been the same.

Doctor Hyatt has directed our special attention to the importance of preserving the molars. He has pointed out to us the result which often follows the loss of the first molar, especially if it is lost after the second molar has taken its place, the tipping forward of that tooth and the loss of occlusion. He has also directed our attention to the painful results where two molars have been lost from the same jaw or mandible. The teeth of the opposite jaw elongate and are often lost as the result of having nothing to oppose them.

Doctor Hyatt's method of anticipating caries in the molars is sound. It is better to take possession of the occlusal surface before caries has attacked it. I have practiced this method for a good many years, but perhaps not quite as radically as Doctor Hyatt does. It is my practice to probe the sulci in the molars as soon as they are erupted, or as soon as the child is placed in my hands, and if the finest probe will enter the fissure, I cut it out with a small bur and fill it after the following method: After it has been prepared, I swab it with a saturated solution of silver nitrate and then fill with Doctor Ames' black phosphate of copper cement. Let me tell you just how I prepare for it. A piece of rubber dam with just a suggestion of vaseline smeared upon its surface is laid on the tray within easy reach. My assistant prepares the cement, the cavity is wiped dry of the silver nitrate. After the cement has been worked into all the sulci I draw my index finger over the rubber dam that has the film of vaseline upon it and press with my finger the cement into every part of the occlusal surface. The cement soon hardens and the excess is removed. I consider that surface safe for a long time to come.

It has been said that the Catholic church asserts that, if it can have the training of the child until it is twelve years of age, they feel confident that no future influence will change its religious belief. If I can have the care of a child's mouth from its birth until twelve or fifteen years of age, I feel that its teeth will be preserved for life.

I feel that the Academy should be most grateful to Doctor Hyatt for his valuable paper.

Dr. I. N. Broomell. *Mr. Chairman, Members of the Academy:* Doctor Hyatt's graphic description of the mythical swamp calls to my mind a statement made a few years ago by our venerable friend, Dr. W. W. Keen. Doctor Keen was addressing a medical meeting and was inclined to find fault with the medical profession because of their shortcomings, adding strength to his remarks by saying that year after year, and decade after decade, the medical profession had been going on treating the so-called diseases of children without any thought or without any effort toward the prevention of these diseases. In a measure it seems to me this remark applies to the dental profession at the present time. True it is we are approaching an age where prophylaxis in dentistry appears to be a very important factor. Doctor Hyatt began his remarks with a preamble in the nature of an apology, saying that it was usual to expect an essayist to present something new. While it is usually the desire of any scientific body to have new things presented, it is not always expected that this will happen. Tonight the unusual thing has happened and Doctor Hyatt has presented in a sense something new.

While the practice of cutting out and filling in the grooves of development in young and newly-erupted teeth is not exactly new, and while Bonwill years ago followed in a measure a similar practice for the same purpose, although in his work he cut away the approximating surfaces of the teeth instead of the occlusal surfaces, doing this with the same idea in view of preventing decay on these surfaces, Doctor Hyatt has brought the subject to us tonight in an entirely new form. He has dignified the operation by giving it an appropriate name. He has furnished in tabulated form, arguments which are sufficient to make us almost pass the resolutions which I understand he has not as yet presented. One of the best features about the paper is the fact that Doctor Hyatt does not want it stored away, or filed in the archives of the Academy whereby it may never again see the light of day. In place of this he wishes something tangible to happen from his efforts and I am sorry that he did not present and ask our action upon the resolutions, so carefully prepared and which I have already read.

I think we can all agree with the major portion of the paper. Those parts which refer to the need of better prophylactic methods, the question of diet and its influence over the calcifying process in the teeth, the question of malnutrition and its detrimental influence over tooth calcification must be accepted without argument. It leaves, therefore, little for discussion. The only thing we have to consider is the advisability of the wholesale cutting into the occlusal surfaces of the cuspidate teeth, with the idea of anticipating future trouble from caries. I have frequently followed this procedure in my own practice and in a few instances have kept a record which has been satisfactory in almost every case. I have by no means made it a universal practice. I scarcely know whether to unqualifiedly support everything which Doctor Hyatt has said or not. There are so many things to be considered. In private practice I doubt the feasibility of it, especially as the generally accepted practice, because there is the question of the management of the child, the co-operation of the parent and the education of both the parent and the child, which from my point of view might disturb the relationship so essential to success existing between all parties concerned. If there is a place for this work—and I believe there is—that place is in the clinic connected with a public institution, in schools and in municipal work. If the question were put to me in regard to passing resolutions favoring this as a wholesale practice to be applied to public clinics such as I have mentioned, then I would be heartily in favor of it.

The question of malnutrition undoubtedly has a very detrimental influence over the development process in the teeth, so far as calcification is concerned, but I am inclined to believe that perhaps many teeth suffer because of the fact that they are erupted too early, this being a result of malnutrition, or lack of nutrition during the time of development. We should also consider the time at which the tooth erupts; this is a very important consideration. We are all aware of the fact that teeth that erupt prematurely show signs of lack of development, or lack of coalescence between the separate lobes of development, while those teeth which are delayed in eruption, are as a class more fully calcified and the coalescence of cusps has taken place completely. The slides were somewhat disappointing to me, because they appeared like selected specimens. In no case did they show what I would classify as a fully formed tooth. Of course I understand that the 74 per cent. of cases referred to are all teeth not fully formed. Before the Academy takes the usual time-honored action of passing the vote of thanks to Doctor Hyatt, I want to have the privilege of doing this myself for this excellent, entertaining and instructive paper.

Dr. J. Leon Williams, New York. The paper to which you have just had the pleasure of listening contains, in my opinion, one of the most important propositions ever made to the dental profession. There is no question in my mind but that Doctor Hyatt's suggestion, if carried into effect, would result in incalculable benefit to humanity. During the later years of my practice in London I followed this method in all mouths which exhibited rapid decay, whenever I could persuade my patients to be so treated, and I have the consciousness today that this was the best professional service I have ever rendered.

My decision to follow this method of treatment in mouths which showed rapid decay was based entirely on the study of the early stages of decay under the microscope.

In a paper read before the New York Odontological Society on January 12, 1897, I called attention to the fact, as shown in photomicrography, that the phenomena of tooth caries penetrated through the entire thickness of enamel and dentin before there was the slightest indication to the unaided eye that there was any defect on the surface of the enamel.

During the past two years I have been over this whole ground again employing new methods of staining, and other new technical methods, and the results are recorded in several hundred photographs. The few comments that I have to make in support of Doctor Hyatt's position are based entirely on these later researches.

And the first thing I wish to say is that Doctor Hyatt has somewhat weakened his own position by speaking of "cutting into sound enamel." If by "sound enamel" we mean perfectly calcified enamel tissue, then I have to say that in all the specimens I have prepared during fifty years' study of the subject I have never found a tooth in which the enamel was perfectly calcified on the morsal surface. And, I will say further, if by perfect calcification we mean enamel that is impervious to fluid, then I do not believe that there is any such thing as perfect calcification on any surface of any tooth. This may sound rather radical to many of you but the statement is backed up by an overwhelming array of facts. Dental caries is not, primarily, the result of defective tooth structure. Let that fact be repeated until it is more fully recognized than it is today. Defective structure is a *predisposing* cause, but in the absence of the active or exciting cause very defective teeth will not decay.

In one of the last papers which Doctor Black wrote he said: "We can not stain the body of normal enamel with any staining agent we know." I said substantially the same thing many years ago, but I know now that I was mistaken. I have many mounted sections of enamel, ground from the finest specimens of teeth that I can find, which are deeply stained with nitrate of silver solution. In nearly all of these specimens the teeth were stained in bulk before being ground. The rapidity with which the stain will penetrate the enamel varies greatly in different teeth, but forty-eight hours is usually a sufficient length of time for this staining agent to completely penetrate the finest enamel. It may be well to note, in passing, that these results completely confirm the physical experiments on the permeability of enamel as reported by Professor William J. Gies.

Now, it seems to me the important point in this connection is this: It is highly probable that the acid of decay will slowly penetrate these channels which are present in all teeth, especially near the surface of the enamel. In nearly all instances it is found that the stain penetrates much more rapidly on the masticating surface of teeth than elsewhere, except in those cases where the acid of decay has already penetrated the proximal surfaces.

It is, therefore, the most certain and sensible form of insurance and a sound economic proposition for the patient to have the defective structure, which always exists on the masticating surfaces of the teeth, cut out and filled as soon after the eruption of the teeth as possible.

The plea that the teeth may not decay and that the patient should have the benefit of the doubt applies, with much greater force, to all forms of insurance, because the chances that the teeth will decay are greater than the risks in any other form of insurance that I know anything about.

In view of this evidence and the statistical facts which Doctor Hyatt has presented in his paper I can see no room for two opinions on the subject. His position in recommending treatment of only the masticating surfaces of molars and bicuspids may be accepted as truly conservative in both senses in which that term is used.

Dr. William A. Jaquette. The Academy is certainly very fortunate in having this matter presented by Doctor Hyatt tonight. He has brought before us the most important subject in dentistry. It is not difficult to get an audience to hear some new principle of bridge work or canal work, or what not, but this big audience tonight is evidence that the profession of Philadelphia recognizes this to be the most important subject in dentistry. When the dentist is given his diploma or license to practice dentistry, he is given his opportunity or privilege of practicing. With that is carried a responsibility, the care of the people who have intrusted themselves and their children to him. Doctor McCollom, I believe, stated in the public press last July that the care of the teeth is a prenatal and preschool problem primarily. We know that in first permanent molars calcification begins at the eighth month of uterine life and that the calcification of all the permanent teeth is largely completed before the child enters school; and this, I may say, is unfortunately as early as many children are first taken to the dentist. Now this question of the percentage. Doctor Hyatt has given in one of the groups, 93 per cent. of the first permanent molars showed carious destruction of the occlusal surfaces. Now we in our offices assume that 93 per cent., or even a lower per cent., is a sufficient warrant for us to take 100 per cent. and treat them in a preventive way. A number of years ago many of you heard Doctor Darby describe his method of so caring for teeth using silver nitrate and copper cement. He has again given it to us tonight. The only point on which I hesitate to support Doctor Hyatt is the extreme question of operating on teeth without caries. In his paper in several places he has described treating these teeth, cutting them *before* they decay. Doctor Darby said that after a very careful examination with the minute point of a piano-wire explorer he would decide whether this is a tooth which must be cut or treated in a purely preventive way. This seems to be the better plan.

I should like to speak a word about explorers. If you visit your dentist friends and ask them to show you their exploring instruments you will find many using a broken excavator or an explorer so thick that it has no spring. That is a very different instrument from the one Doctor Darby has described, the piano-wire explorer. If you do not know it, get acquainted with it. Doctor Hyatt says when a tooth is erupted he protects it *before* it has decayed, with a silver cement or the oxyphosphate of copper. That carries the tooth for a number of years, or such time until he can put the rubber dam on and cut it out. Now, if he has carried that tooth through that most susceptible time, why not renew the oxyphosphate of copper or silver cement for another period of time? The time of greatest susceptibility of a tooth is from its first eruption. It takes some months and sometimes years before teeth are in actual occlusion, when the stress of mastication polishes the surfaces. The slides of Doctor Williams show the penetration of silver nitrate. I am not in position to say what three minutes' application of silver nitrate would do; I would therefore hesitate to adopt this extreme measure of operating on all the teeth where I can find no failure of fusion of enamel rods. Doctor Broomell has said that he would like to see this tried in clinics. I would like him to recall the examination of children's teeth in clinics and private practice. I confess to surprise when I found teeth in the children of families who come to me often more perverted than those of the children in the clinics. I went with Doctor Fones in Bridgeport to a school made up almost entirely of foreigners, many of them Italians. Now in the diet of these children in Italy there is no free sugar except in the cities. The only sugar the children get is in the fruit which is eaten, for often they have no cane or artificial sugar. The teeth of these children in Bridgeport, children of foreign settlements, are the best teeth I have ever seen.

Dr. O. G. L. Lewis. I have listened with a great deal of interest to this paper by Doctor Hyatt and it is, as

Doctor Darby and Doctor Jaquette have said, one that is of vital interest to us all. I wonder how many of us recall our dental history and know where were recorded the first writings in reference to preventive dentistry? I wonder how many of us recall that the first reference to the care and cleaning of the teeth is in the Ebers papyrus, written thirty-seven centuries before Christ and today, fifty-six centuries later, we are talking about the same thing. How many of us recall that Hippocrates, who lived about 450 B. C., spoke of the prevention of the disease by treating it at its origin, which is nothing more or less than Doctor Hyatt has given us today; good gospel given centuries ago, which the profession has failed to follow. I am glad that he did not claim that it was anything new, for it is nothing more or less than was published in a little booklet in Baltimore in 1867 by Robert Arthur. In this book in speaking of treating the occlusal surfaces of molars he refers to the presence of decay in the fissures, and says: "Decay or decomposition at such point is usually inevitable; no care short of the obliteration of these fissures can prevent its occurrence. The only manner in which this can be accomplished is by the enlargement of the fissures, unless this has already occurred as a consequence of decay, and the formation of cavities which will securely retain some substance capable of resisting decay." The trouble with the profession is that they did not accept Doctor Arthur's gospel. Let us as a body of men and women here tonight make up our minds that we are going to follow the gospel which has been preached by these certain men for fifty-six centuries. In my own practice it has been used for nearly twenty years. I heard Doctor Darby speak of this in class, and later saw Doctor Perry demonstrate with beautiful instruments, so small and minute that the average man could not operate with them, first sterilizing the fissures and then packing them with tin to prevent caries. As Doctor Jaquette said, I can not agree with the extreme cutting. I believe if the premolars and molars are taken at the age when first erupted and even before they are entirely through, when we can push back the gum, dry thoroughly with alcohol and fill; these teeth can be held until they come into occlusion with the teeth of the opposing jaw. After this a very small percentage of them will decay. I believe if I can hold these surfaces until the teeth come into their full occlusion, that I have accomplished a great deal, and that from then on particular attention should be given to proximal surfaces, as the occlusal surfaces will be protected to a great extent by the constant grinding of food upon them. I want to compliment and thank Doctor Hyatt for bringing this subject here and giving it to us as it has been given. It is splendid gospel and if followed will help us to save many teeth. Thank you!

Dr. Stirling Hewitt. The Maoris had the most perfect teeth. Three generations of the public schools of New Zealand the English have taken care of the Maori children and they show pretty much the same condition of teeth as children in our public schools. Whether that is due to diet or lack of exercise I hesitate to say, but I think it requires something more than diet. We require some operative prophylactic measures, and for a long time I have been using a method similar to that of which Doctor Jaquette and Doctor Darby spoke.

Dr. V. Pinnock Bailey. I wish to add my own to the many compliments already expressed to Doctor Hyatt. I think it is quite a contribution to preventive dentistry. I wish to ask the essayist one or two questions which may be a departure from the paper.

I recall some months ago listening to a very interesting paper read by an essayist in this room, and he dwelt extensively on the diet. I realized from his speech that the form of diet which consisted largely of bread, meat and potatoes, was the life-giving constituent of each meal, and I have been comparing that with Doctor Hyatt's statement that in the various clinics he has attended he noticed the

Italians had considerably less decay than one would find in the average child in this country. I wish to indorse that part, as I find in my experience that, in the children of Italian (foreign) parentage and birth, there is small percentage of decay existing; whereas, the child born in this country of Italian parentage has a very high percentage of decay. In that part of the world the diet consists of little or no meat (particularly cold storage), hence a smaller degree of putrefactive intestinal disturbances.

In the tropical regions where they indulge in the free use of sugar, there is also a small percentage of decay existing in the molars of the child or adult. There possibly it may be due to the various kinds of food the average person uses containing a higher percentage of natural salts, little or no cold-storage meats, and in conjunction with these there is a particular form of herb or stick (its botanical name is "Guanicana") commonly called "chew-stick." It is first chewed and the foam produced is used to cleanse the teeth; its taste is bitterish. The associated factors combined have probably helped prevent decay.

Would the essayist inform me if the condition existing as erosion warrants the necessary care of the teeth from an operative standpoint that he suggests?

Dr. Thaddeus P. Hyatt (closing the discussion). It is gratifying to know from the remarks made by those who discussed my paper that the suggestions offered have been so cordially received.

Impressions may often lead us astray. We may believe there are a far larger number of perfect sets of teeth than there really are. Among the 7,000 employes of the Metropolitan Life Insurance Company, we know of only one set of thirty-two teeth with no cavities. Frankly, had any one asked me if I had seen many sets of perfect teeth, I would have answered, "Yes."

While I have not attempted to present any new thing this evening, I have tried to give to you the actual facts of present conditions as related to decay found on the occlusal surfaces of molars and premolars.

When vaccination as a prevention for smallpox was first offered to the medical profession there were many who did not believe in it. But today we have sufficient data to be able to prove its value and efficiency.

The mortality table in smallpox is lower than the figures I have presented regarding loss of first molars or cavities on occlusal surfaces:

Mortality Table in Smallpox per 1,000

| Vaccinated | Unvaccinated |
|---|---|
| 6.70 | 31.4 |

Smallpox Epidemic in Luxemburg, 1895
Mortality Table per 1,000

| Vaccinated | Unvaccinated |
|---|---|
| 10 | 50 |

Loss per First Molars per 100
47%

Cavities in Occlusal Surface per 100
74%

Doctor Darby has stated that he does not wait for visible signs of decay but excavates and fills just as soon as his explorer sticks. Doctor Williams has shown from microscopic examination that acid penetrates the entire thickness of the enamel before the finest explorer can detect it.

Doctor Eckerman has written a book on "Dental Caries in Relation to Oral Osmosis." Bodecker, Bunting and others are investigating the porosity of teeth. Admitting for the sake of argument that osmosis is a necessary factor in dental caries, it must have its greatest efficiency in the occlusal fissures. It is well known that if we intercept osmotic action, it stops. Therefore if we cut out these fissures and fill with metal we have blocked osmotic action.

Granite has blind joints, places where there has not been a perfect crystallization. These blind joints are susceptible to decay.

Seventy-four per cent. of occlusal fissures on molars and premolars have faulty calcification of the enamel. Doctor

Williams tells us that in fifty years of research work he has never found one tooth that had perfect enamel calcification. This would mean that 100 per cent. of these fissures were faulty. Cutting out this blind joint or imperfect enamel formation, before further destruction has taken place, before bacterial invasion has taken place, would seem a safe and sane procedure to follow.

The dental profession can be excused for not having followed the advice of the early advocates of this procedure, as none of us believed conditions were as bad as they are. But today, with the aid of many organizations gathering figures in different parts of the country, the actual conditions are laid before us.

Can we ignore them?

We should now establish the new order of preventive dentistry and prevent the occurrence of decay. To do this necessitates the adoption of properly worded resolutions by your Academy, and I trust that the presentation of these facts will enable your Society to be the first dental organization to take this step.

### SOME ADDITIONAL REMARKS BY THE AUTHOR

It is most gratifying to know that my efforts to arouse and direct attention to the prevalence of decay upon occlusal surfaces of molars and premolars as compared to the other surfaces of these teeth and the suggested procedure for preventive measures has received so much attention and consideration.

Proper investigation of any proposed procedure must be discussed and viewed from all sides. Opposition is almost always necessary to bring out the faults or virtues of the plan suggested.

My purpose in adding any further remarks is to call attention to what I believe to be illustrations that are not parallel or analogous to the central idea of my paper.

It is very necessary that a clean cut and definite question be presented so that all misunderstanding may be avoided. The question is this:

Whereas, a very large per cent. of all first permanent molars are lost before the age of forty, and

Whereas, seventy-four per cent. of all occlusal pits and fissures in first and second molars and second premolars decay,

Therefore, it will be a wise procedure to cut out these pits and fissures upon the occlusal surfaces and fill before decay has started.

In support of this I have presented figures from over thirty-nine thousand cases from different parts of the country.

Several of those who discussed the paper said they preferred to only cut out fissures into which they could enter a fine probe.

Does it follow that caries is always present when a fissure permits the entrance of a fine probe? I believe that there are many fissures that permit the entrance of a fine probe in which no caries will be found. I believe, however, that it is only a question of time and physical condition when seventy-four per cent. of these pits and fissures will have caries, regardless of mouth conditions.

The Editor of the *Dental Cosmos* has complimented me by writing an editorial upon the subject.

It is with a great deal of diffidence that I call attention to some illustrations used and which I think are not relevant to the subject. I do this in the hope of calling forth further discussion upon the advisability or inadvisability of the procedure advocated.

Attention is called to the theory of Robert Arthur and the irrational system of tooth mutilation. If this illustration is offered to show the dangers of accepting the procedure of cutting out all occlusal fissures before decay has started I do not think it is an analogous case, because

First. The procedure advocated by Arthur called for destruction of tooth surface, which, with care, can be kept clean.

Second. There was no replacement of lost tooth substance with a material not affected by caries.

Third. The theory was not based upon a study of actual conditions, but upon the assumption that interproximal surfaces are more susceptible to decay than other surfaces. This is not true, as figures secured and presented in my paper will show.

Fourth. The formation of the tooth in occlusal fissures is quite different to than on the mesial and distal surfaces, and the same procedure is not called for in both locations.

Fifth. In the treatment advocated for occlusal fissures, not only is there replacement of lost tooth substance with material not affected by caries, but the normal shape and conformation of the tooth are retained.

The next illustration is the statement of Doctor Black, that "caries of the teeth is a factor of the environment of the teeth and not of the structural peculiarities of the teeth themselves."

If caries of the occlusal pits and fissures is a factor of the environment of the teeth and not of the structural formation of these fissures, how is it that we find that caries in the occlusal fissures doubles the number of cavities found in all the other four surfaces added together?

Did Doctor Black have reference to occlusal fissures when he made this statement, or to the structural peculiarities of the entire tooth?

I ask this because Doctor Black has also stated that pits and fissures give opportunities for causes that induce caries. These two positions are entirely different, and while it may be true that structural peculiarities of the entire tooth as to its being hard or soft do not make it more susceptible to decay, pits and fissures because of their shape and formation give greater opportunity for the causes of decay to work.

While I believe we must look for two factors as the probable cause of decay, and not only one, I have intentionally refrained from any consideration of this aspect of the question. As stated in my paper, it was not my idea that the procedure advocated would prove the panacea for the prevention of all dental troubles, but that it might tide us over to that period when a better understanding of prenatal care, nutrition and kindred subjects would make such work no longer necessary.

To refrain from advocating a procedure because some would abuse it would stop the introduction of all advancement. I fully appreciate the dangers that the empiric and unthinking element of our profession might do, but I frankly question if they could cause more damage than is being done now by orthodox dental caries, particularly when we remember the simplicity of the operation, the replacement of lost tooth substance and the restoration of normal tooth shape, or cause the loss of more teeth than are being lost now by our present neglect of preventive measures.

In conclusion, may I ask those who are interested and believe that preventive measures are possible and that something should be done for the countless millions of erupting molars that can never receive the benefits of the new knowledge of nutrition, to co-operate and discuss the practicability or impracticability of the procedure here advocated. As an aid for your consideration I enclose a reprint of the paper with the discussions and editorial included.

